# Contact with dogs is associated with improved survival in cancer patients

**DOI:** 10.1038/s41598-026-39952-z

**Published:** 2026-02-17

**Authors:** Robert Preissner, Zhengjie Yang, Saskia Preissner, Christa Thöne-Reineke

**Affiliations:** 1 Institute for Animal Welfare, Animal Behavior and Laboratory Animal Science, Freie Universität Berlin, Berlin, Germany; 2 Institute of Physiology and Science-IT, Charité - Universitätsmedizin Berlin, Philippstr. 12, 10115 Berlin, Germany; 3 Oral and Maxillofacial Surgery, Charité - Universitätsmedizin Berlin, Philippstr. 12, 10115 Berlin, Germany

**Keywords:** Cancer, Dog ownership, Prognosis, Survival rate, Real-world evidence, Cancer, Diseases, Medical research, Oncology, Risk factors

## Abstract

For cardiovascular diseases, diabetes, and asthma, the positive effects of dog ownership are shown. Cancer is a leading cause of death, but the influence of dogs on cancer incidence and survival is not well examined. As modifiable lifestyle factors gain importance in cancer survivorship research, the potential protective role of dog ownership warrants systematic investigation. We retrospectively analyzed clinical data from a federated global health research network, focusing on patients diagnosed with cancer (International Classification of Diseases (ICD-10): C00-D49). From these, we generated two cohorts with contact with dogs (cohort 1) and one without (cohort 2). After propensity score matching for age and sex, a total of about 55,000 patients were included. Analysis of the matched cohort demonstrated that dog ownership was significantly associated with reduced 5-year all-cause mortality in cancer patients compared to non-owners (RR = 0.44). Survival analysis revealed a significantly higher 5-year cumulative survival rate among dog-owning patients versus non-owners, with a hazard ratio (HR) of 0.36. Contact with dogs is associated with a 64% relative risk reduction in cancer mortality, potentially mediated by increased physical activity, psychosocial support, and microbiome modulation. While retrospective design precludes causal inference, this first large-scale matched cohort study provides compelling epidemiological evidence warranting prospective validation.

## Introduction

 Cancer remains a significant threat to human longevity, and its prognosis has been a key focus of extensive research^1^. In recent years, advancements in chronic disease management models have drawn increasing attention to the role of modifiable lifestyle and psychosocial factors in the long-term survival of cancer patients^2,3^. Studies indicate that factors such as physical activity levels, stress regulation capacity, and social support networks may influence survival outcomes through various bio-psycho-social mechanisms^4–8^.

Against this backdrop, pet dog ownership has increasingly been recognized as a potential protective factor in research. The latest epidemiological survey in the United States shows that household dog ownership has risen from 37.5% in 2012 to 45.5% in 2024, reflecting a growing societal interest in the health benefits of companion animals^9^. Evidence suggests that dog ownership enhances owners’ health through various mechanisms, including promoting light physical activity, reducing loneliness and social isolation, and alleviating anxiety and depressive symptoms^10–12^. Notably, these benefits closely align with key factors in cancer prognosis management.

The clinical benefits of animal-assisted interventions have been preliminarily validated across various disease domains. Cohort studies have shown a significant association between dog ownership and a reduced incidence of hypertension, diabetes, and cerebrovascular events in the general population, with the protective effect remaining statistically significant after adjusting for confounding factors^13,14^. In chronic disease prevention, observational studies suggest that dog ownership is linked to a lower incidence of asthma and allergic rhinitis and may contribute to regulating type 2 diabetes risk, though findings in these areas remain inconclusive^14,15^.

Further cohort study evidence indicates that dog ownership is significantly associated with a reduced risk of rehospitalization and all-cause mortality among myocardial infarction survivors^16^. Similarly, in patients with ischemic stroke, dog ownership has been statistically correlated with a lower risk of all-cause mortality^16^. However, in oncology, substantial evidence gaps persist. Although small-sample studies on specific cancer types, such as breast and prostate cancer, have suggested that dog ownership may enhance patients’ physical activity levels and thereby improve prognosis, retrospective analyses of lung cancer have shown no significant impact of dog ownership on mortality rates^10,17^. These conflicting findings highlight the urgent need for large-scale studies to clarify the relationship between dog ownership and cancer outcomes.

It is important to note that current studies on this topic often face methodological limitations, such as insufficient sample representativeness and limited statistical power. To systematically assess the association between dog ownership and cancer prognosis, this study constructed a retrospective cohort using a large real-world database to examine the relationship between dog ownership and 5-year mortality in cancer patients. Our research hypothesis suggests that cancer patients exposed to dogs (i.e., dog owners) have a higher 5-year survival rate compared to those without dog exposure, indicating that dog ownership may be associated with a reduced risk of mortality within 5 years.

## Methods

### Ethical approval

The study utilized electronic health records from the TriNetX platform for retrospective analysis, without active interventions or direct patient contact, thereby qualifying for an exemption from informed consent under the Health Insurance Portability and Accountability Act (HIPAA) for retrospective observational studies. The patient dataset was de-identified by a specialized team in strict compliance with HIPAA § 164.514(a) and (b)(1), ensuring that participants’ identities remained untraceable. The most recent compliance verification, conducted in December 2023, confirmed adherence to regulatory requirements. This study followed international ethical guidelines for observational epidemiological research and secondary clinical data analysis, maintaining compliance with the ethical framework governing medical data usage in accordance with the Declaration of Helsinki. This research was declared as non-human subject research and is therefore exempt from ethical approval and informed consent (Ethikkommission der Charité Universitätsmedizin Berlin).

### Cohort definition

This study is a retrospective cohort analysis based on the TriNetX global healthcare data platform, utilizing multi-center electronic health records (EHRs) to examine the association between dog exposure and cancer prognosis. Data collection was completed on August 8, 2024, with the study population restricted to hospitalized cancer patients (ICD-10: C00-D49). The dog exposure group included patients diagnosed with cancer who had a documented history of dog exposure (ICD-10: W54), while the non-exposure group comprised hospitalized cancer patients without any recorded dog exposure. The date of the first hospitalization with a cancer diagnosis was designated as the baseline time point for all enrolled cases. Additionally, patients in the dog exposure group were required to have documented exposure before this baseline. Although the study protocol initially specified the exclusion of cases with a baseline exceeding 20 years, no such cases were present in the dataset.

**Fig. 1:**
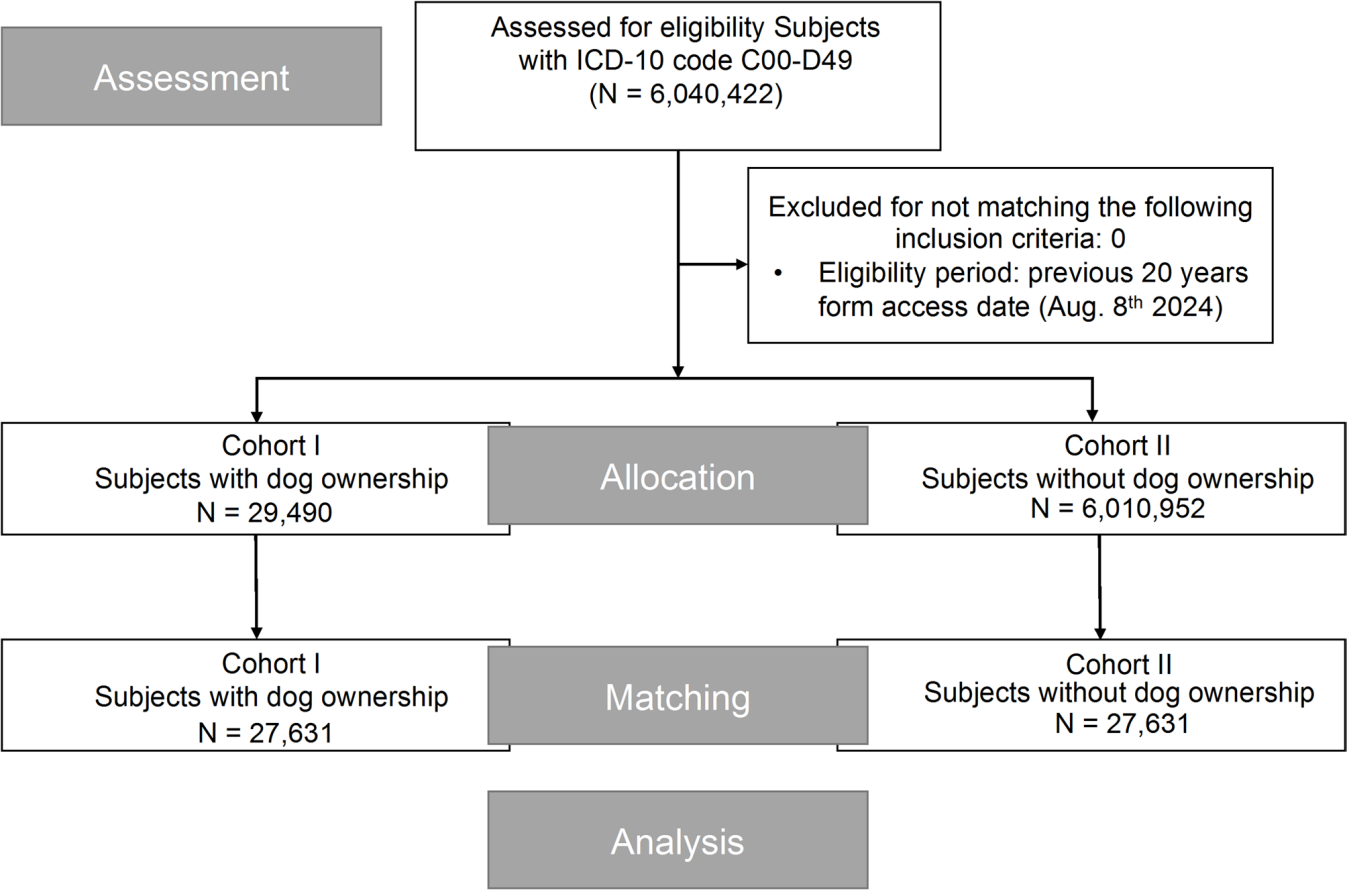
CONSORT flow diagram illustrating the data selection and matching process for cancer patients with and without dog ownership.

Before propensity score matching (PSM), the initial cohort consisted of 29,490 patients in the dog exposure group and 6,010,952 patients in the control group. To adjust for baseline differences in age and sex, logistic regression was used to generate propensity scores, followed by 1:1 nearest neighbor matching (NNM) with a caliper of 0.01. The final matched cohort included 27,631 patients in each group, achieving a good balance across covariates. The mean age (52.1 ± 19.3 years) and sex distribution (60.8% female) were well-matched, as indicated by a standardized difference of < 0.1 and a *p* value of 1.0, ensuring the validity of subsequent survival analyses (Fig. 1). Notably, no explicit age restrictions were applied at enrollment. The higher mean age observed in the exposed group may reflect the preferential capture of older hospitalized patients who had both a documented neoplasm diagnosis and recorded dog contact.

### Statistical analysis

 This study employs the 5-year all-cause mortality rate as the primary outcome measure and systematically assesses the impact of dog ownership on the survival outcomes of cancer patients using a propensity score-matched cohort. In the association analysis phase, we quantified mortality differences between exposed and unexposed groups by calculating the risk difference (RD), risk ratio (RR), and odds ratio (OR). Additionally, we reported the statistical significance of group differences along with their corresponding 95% confidence intervals (CI). For survival analysis, we applied the Kaplan–Meier method to generate survival probability curves for both groups and used the log-rank test to evaluate the statistical significance of intergroup differences. To further examine the dynamic effect of dog ownership on mortality risk, we employed a Cox proportional hazards regression model to estimate the hazard ratio (HR) and its 95% CI. Patients lost to follow-up or who withdrew during the study period were treated as right-censored, with the censoring time defined as the date of their last recorded visit. The analytical time window commenced on the first day after cancer diagnosis (index event) and extended until either a death event occurred or the 5-year follow-up period was completed, whichever came first, ensuring full coverage of all endpoint events within the follow-up period.

## Results

 The initial cohort of this study comprised 6,040,422 cancer patients, including 29,490 patients in the dog ownership group (Cohort 1) and 6,010,952 patients in the non-dog ownership group (Cohort 2). Due to significant differences in baseline characteristics, specifically age and sex, between the two groups, propensity score matching (PSM) was conducted at a 1:1 ratio, resulting in a balanced cohort of 27,631 matched pairs. Following matching, the age distribution (52.1 ± 19.3 years) and sex distribution (60.8% female) were identical between the groups (*P* = 1.0, see Table 1).

**Table 1:** Cohort characteristics before and after propensity score matching.

	Before matching				After matching			
	Cohort 1 *N* = 29,490	Cohort 2 *N* = 6,010,952	*p* value	Std diff.	Cohort 1 *N* = 27,631	Cohort 2 *N* = 27,631	*p* value	Std diff.
Age at index	52.1 ± 19.3	57.0 ± 19.1	< 0.001	0.253	52.2 ± 21.2	52.2 ± 21.2	1	< 0.001
Females	16,793 (60.8%)	2,969,701 (54.6%)	< 0.001	0.125	16,793 (60.8%)	16,793 (60.8%)	1	< 0.001

Association analysis revealed that the 5-year mortality rate in the dog ownership group was 4.2% (1,164/27,631), which was 5.4% points lower than the 9.6% (2,646/27,631) observed in the non-dog ownership group (95% CI − 0.058 to − 0.049, *P* < 0.001). Cancer patients who owned dogs exhibited a 56% lower risk of death within 5 years compared to those without dogs (HR = 0.44, 95% CI 0.41–0.47, Table 2).

**Table 2:** Summary of risk analysis for death and hospitalization in cohort 1 and cohort 2.

Outcome	Cohort	Events (*n*)	Risk	Risk difference (95% CI)	*p* value	Risk ratio (95% CI)	Odds ratio (95% CI)
Death	1	1164	0.042	− 0.054 (− 0.059, − 0.049)	< 0.001	0.440 (0.411, 0.470)	0.415 (0.387, 0.446)
	2	2646	0.096				

**Fig. 2:**
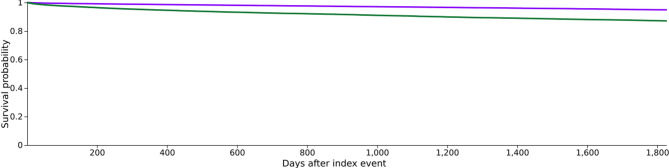
Kaplan–Meier curve (5-year survival) for cancer patients with (purple) and without (green) dog ownership.

In the survival analysis, Kaplan–Meier curves show a significantly higher 5-year cumulative survival rate in the dog ownership group (94.89%) compared to the non-dog ownership group (87.12%), with a statistically significant difference (Log-Rank test: χ^2^ = 913.787, *P* < 0.001, Fig. 2). Furthermore, compared to non-dog owners, cancer patients who owned dogs experienced a 64% reduction in the cumulative all-cause mortality risk over 5 years (HR = 0.361, 95% CI 0.337–0.386, *P* < 0.001, Table 3).

**Table 3:** Kaplan–Meier survival analysis.

Cohort	Patients in cohort	Patients with outcome	Median survival (days)	Survival probability at end of time window
1	27,631	1164	–	94.89%
2	27,631	2646	–	87.12%

Log-rank test	χ^2^	Df	*p* value
	913.787	1	< 0.001

Hazard Ratio and Proportionality	Patients in cohortPatients in cohort	χ^2^	df	*p* value
	0.361 (0.337, 0.386)	65.995	1	< 0.001

## Discussion

This study explores the potential impact of dog ownership on 5-year survival outcomes in cancer patients. Previous research suggests that dog ownership may promote mild physical activity, enhance social support, and improve lifestyle behaviors. Based on these findings, we hypothesize that dog ownership is associated with a reduced risk of all-cause mortality in patients with cancer. We employed PSM to achieve a 1:1 match between dog owners and non-dog owners based on age and sex. Our results indicate that cancer patients who owned dogs had a significantly lower 5-year mortality risk compared to those who did not (risk ratio, RR = 0.44). Furthermore, their 5-year cumulative survival rate was markedly higher (94.89% vs. 87.12%; HR = 0.361; *p* < 0.001). This study is the first to use a large, matched cohort to provide epidemiological evidence suggesting that dog ownership may serve as a protective factor for improving cancer patient survival.

The protective effect of dog ownership on cancer patients may be mediated through multiple factors. Surveys of cancer survivors indicate that dog walking significantly increases daily light physical activity^5^. This sustained activity can enhance cardiopulmonary function and reduce muscle atrophy, ultimately improving quality of life and potentially influencing survival outcomes^18^. Additionally, studies highlight the role of canine companionship in alleviating depressive symptoms, reducing loneliness, and mitigating social isolation, providing psychological and social explanations for the observed protective effect of dog ownership on 5-year survival rates in cancer patients^12,19,20^. The cancer treatment process often involves psychological challenges such as depression and anxiety, which are linked to poor prognosis^21–23^. The unconditional companionship and emotional support/motivation provided by pets may help reduce loneliness and social isolation, fostering a more positive outlook on illness. Improvements in psychological well-being may indirectly enhance cancer survival by mitigating adverse neuroendocrine responses and promoting treatment adherence, further supporting the potential role of dog ownership as a protective factor in cancer prognosis^24^.

Beyond physical activity and psychological factors, dog ownership may influence cancer patients’ survival outcomes by altering gut microbiota. The human and canine gut microbiomes share significant homology, suggesting that dog owners may acquire beneficial microorganisms from their pets^25^. Recent research supports this hypothesis, showing that dog owners exhibit a significantly higher abundance of beneficial bacterial taxa, such as *Bifidobacteriaceae* and *Ruminococcaceae*, compared to non-dog owners^26^. These microbes may improve cancer prognosis through multiple mechanisms, including immune modulation, short-chain fatty acid production, gut homeostasis maintenance, and chronic inflammation reduction—key factors in inhibiting tumor progression^27,28^.

This study has several limitations. First, as a retrospective observational study, it does not allow for direct causal inference. Although PSM balanced age and sex, unmeasured confounders such as socioeconomic status, cancer stage, and treatment regimens may still have influenced the results. For instance, severe cancer cases with a bad prognosis might have less contact with dogs (physical frailty, immunotherapy, etc.). Second, because our data were derived from EHRs, we used the ICD-10 diagnosis code as a proxy for exposure to dog ownership. This code captures only dog-related exposure events that are documented in medical records and cannot provide more granular exposure information, such as the duration of dog contact, number of dogs owned, daily companionship time, whether the patient was the primary caregiver, or other factors that may influence the strength of the bond between human and dogs. These unmeasured differences may introduce heterogeneity at the “dose-response” level, thereby affecting the outcome. Additionally, some patients without dog ownership may have been exposed to pets through other means, such as visiting others’ pets or participating in animal-assisted therapy programs. These measurement errors may have led to either an underestimation or an overestimation of the true effect. Moreover, the analysis did not fully account for other lifestyle factors, such as diet, smoking, and alcohol consumption, due to partial missingness in EHR data. Ensuring data completeness could have reduced the sample size and weakened statistical power. Therefore, the protective effect observed in this study should be interpreted with caution. Future research should utilize large-scale, prospective cohort studies to clarify the temporal relationship between dog ownership and cancer survival outcomes. Additionally, given the heterogeneity across different cancer types, stages, and treatment modalities, stratified analyses are essential to further elucidate the association between dog ownership and cancer prognosis. Fourth, in defining the cohort, we restricted the study population to hospitalized patients with neoplasms. This decision was primarily driven by feasibility considerations: in routine clinical practice, outpatient oncology records often do not systematically capture lifestyle factors such as dog ownership and pet contact, whereas inpatient records typically contain more detailed medical histories and exposure-related documentation. In addition, follow-up and outcome ascertainment in EHRs, such as mortality information, is generally more complete among hospitalized patients. Accordingly, the generalizability of our findings is limited and should not be directly extrapolated to patients with cancer who were never hospitalized and were managed exclusively in outpatient settings; the association in that population warrants further investigation using more appropriate cohort definitions and data structures. Finally, although we did not impose explicit age restrictions at enrollment, the age distribution of our sample skewed older, which further limits extrapolation to pediatric and adolescent oncology patients. The relationship between dog ownership and cancer prognosis in children and adolescents should be evaluated in dedicated pediatric cohorts with more stringent eligibility criteria and more precise exposure assessment.

## Conclusion

This retrospective analysis, based on a global multicenter medical database, found that cancer patients who had contact with dogs have a higher 5-year survival rate than those who did not, suggesting a potential protective effect of dog ownership against cancer-related mortality. Increased physical activity, psychological support, and possible immunological benefits associated with dog ownership are considered key factors in this improved survival. Further prospective studies are needed to confirm the causal relationship and clarify the underlying biological mechanisms. If validated, pet companionship could serve as a valuable adjunct to comprehensive cancer care, with significant implications for enhancing patient prognosis and quality of life.

## Data Availability

Data is provided within the manuscript. Raw data can be retrieved by the corresponding author upon request.
